# Binge Drinking and Obesity-Related Eating: The Moderating Roles of the Eating Broadcast Viewing Experience among Korean Adults

**DOI:** 10.3390/ijerph18158066

**Published:** 2021-07-30

**Authors:** Jiye Kim, Saegyeol Choi, Hyekyeong Kim, Soontae An

**Affiliations:** 1 Department of Health Convergence, Ewha Womans University, 52, Ewhayeodae-gil, Seodaemun-gu, Seoul 03760, Korea; yd7470@ewhain.net (J.K.); new_wave@ewhain.net (S.C.); 2 Department of Communication and Media, Ewha Womans University, 52, Ewhayeodae-gil, Seodaemun-gu, Seoul 03760, Korea; soontae@ewha.ac.kr

**Keywords:** binge drinking, eating broadcast, obesity-related eating behavior, external eating, women, twenties

## Abstract

Recently, there has been a notable rise in binge drinking and in the popularity of eating broadcasts via TV and online platforms, especially in Korea. This study analyzed the moderating effect of the eating broadcast viewing experience on the relationship between binge drinking and obesity-related eating behaviors. Cross-sectional self-reported online survey data were collected from 1125 Korean adults. Moderation models for restrained, emotional, and external eating behaviors were tested using moderation analyses with Hayes’s PROCESS version 3.5 compatible with SPSS. As a result, the eating broadcast viewing experience moderated the relationship between binge drinking frequency and external eating (F_change_ = 2.686, *p* = 0.045). More frequent binge drinking was associated with a higher level of external eating in participants who only watched online eating broadcasts, especially among women. Participants in their twenties showed the same above association; additionally, those who only watched TV eating broadcasts showed an inverse association, indicating that more frequent binge drinking was associated with a lower level of external eating. Consequently, an eating broadcast viewing experience was one of the environmental factors associated with binge drinking that influences obesity-related eating behaviors.

## 1. Introduction

In 2020, the total annual alcohol consumption per capita in those aged 15 and older in the Organisation for Economic Cooperation and Development (OECD) was 8.8 L of pure alcohol, whereas in Korea it was 8.5 L [1]. However, the monthly binge-drinking rate among Korean adults was 38.7% in 2019, rising to 48% for those in their 20s, which has remained stable over the previous decade [2]. The term binge drinking typically means the consumption of seven or more drinks for a male or five or more drinks for a female within a single day [3]. Binge drinking is a serious societal problem and a risk factor for many chronic diseases, such as cardiovascular disease or liver disease [4,5]. Moreover, a systematic review discovered positive associations between alcohol intake and the risk of weight gain in studies with data on higher levels of drinking [6]. Among Korean adults, binge and heavy drinking were linked to an increased risk of obesity or metabolic syndrome [7,8].

Binge drinking influenced individual obesity-related eating habits, such as increased food consumption and binge eating, by amplifying people’s perception of appetite [9,10]. Certain behaviors, such as binge drinking and obesity-related eating, could be affected by socio-cultural and environmental factors [11]. Binge drinking behavior was specifically associated with parents, peers, and the social environment, which included drinking norms, alcohol policy, traditional drinking patterns, and social media [12]. In the context of society, culture, and environment, it is necessary to determine how binge drinking affects obesity-related eating behaviors.

Among environmental factors, the media environment, including mass media and social media, is a major factor in Korea. An eating broadcast about eating or cooking food, known as “Mukbang”, has recently become popular in Korea. This eating broadcast began in Korea and is now spreading all over the world [13]. These eating broadcasts show various types of food and eating behaviors and are posted in a format that can be accessed by anyone, regardless of age [14,15]. However, since previous studies on eating broadcasts focused mostly on cultural and psychological analysis, there is a lack of research on the impact of watching eating broadcasts on health problems and behaviors [13,14,16,17]. We investigated the health problems caused by watching eating broadcasts concerning binge drinking.

The eating broadcasts began on a personal internet broadcasting website, but have appeared on mainstream media, such as television [18]. Rapidly emerging interactive digital social media channels, such as online platforms, combined with traditional mass media such as television, characterize the current media environment [19]. Eating broadcasts on new online media platforms are more popular than those on television (TV) due to the unity between content providers and consumers based on their dailiness and empathy [20]. An increase in one-person households and the resulting loneliness, as well as the growth of one-person media broadcasting through smartphones, may factor in the popularity of eating content [21]. Since eating broadcasts are expected to continue to grow in popularity and influence, it is necessary to identify the influence of the type of eating broadcast viewing experience.

Previous studies identified the negative effects of mass media use or exposure on unhealthy eating habits and eating disorders [22,23,24,25]. TV exposure was not always directly negative for eating disorder symptoms [26]. Recent social media or internet use affects obesity and eating disorders [27,28,29,30,31]. However, there is a lack of research on the potential impact of eating broadcasts on eating behaviors. There has been no research on how watching eating broadcasts affects problematic eating behaviors in people who have a drinking problem. As a prior study reported a positive association between watching eating broadcasts and eating disorders [32], further relevant exploratory studies are needed.

Therefore, the purpose of this study was to identify the interaction between binge drinking and eating broadcast viewing experience on obesity-related eating behaviors among Korean adults. Also, we aimed to find out which obesity-related eating behavior was affected by the interaction between binge drinking and eating.

## 2. Materials and Methods

### 2.1. Participants and Procedures

A cross-sectional self-reported online questionnaire-based survey was conducted in the second half of 2019. According to Statistics Korea, the total population of Korean adults in 2018 was 42,971,668 [33]. The sample size was calculated at confidence level 95% and confidence interval 3, for the population of 42,971,668, which resulted in the minimum required sample of 1067, however, the sample size was increased to be 1125 considering about 5% of the potential dropout rate.

The subjects of the study were recruited by posting recruitment advertisements on a research website linked to our online survey. Individuals who met all inclusion criteria could be included in this study. Participants were eligible if they were at least 19 years old, lived in South Korea, watched TV shows more than once a month, and watched movies on online platforms more than once a month. Before participating in the online survey, respondents were screened for eligibility with the questions of sex, age, residence, and experiences of media use.

A total of 1125 people were sampled to represent the Korean people by allocating people living in 17 metropolitan and provincial governments by sex, age group, and region. All subjects read a detailed explanation of the study and agreed to participate before responding. Since each participant had their own account with access to the online survey software, no one person could complete the questionnaires more than once. All research procedures and methods were approved by the Institutional Review Board of Ewha Womans University. 

### 2.2. Measures

#### 2.2.1. Demographic Statistics

Sociodemographic characteristics included sex, age, marriage, annual household income, and area of residence. Health status was identified with weight, height, stress level, depression level, and disease history. To diagnose obesity, weight and height were used to calculate body mass index (BMI) using the formula weight/height^2^ (kg/m^2^). According to the WHO’s Asia–Pacific guidelines, less than 18.5 kg/m^2^ was considered underweight, 18.5 kg/m^2^ or more but less than 23.0 kg/m^2^ was considered normal weight, 23.0 kg/m^2^ or more but less than 25.0 kg/m^2^ was considered overweight, and 25.0 kg/m^2^ or more was considered obese [34]. The question, “How much stress do you feel in your daily life?” was used to assess stress levels. This was measured on a five-point (1–5) Likert scale, with 1 meaning, “I don’t feel it at all” and 5 meaning, “I feel it a lot.” Depression level was assessed using the Korean version of the Depression Screening Tool (PHQ-9), a reliable and valid tool consisting of nine four-point (1–4) scale questions about the frequency of depression-related symptoms over the previous two weeks [35]. This study had a Cronbach’s alpha value of 0.870. The sum of the scores was calculated and divided into five categories of symptom severity: 1 was identified as minimal (1–4 points), 2 as mild (5–9 points), 3 as moderate (10–14 points), 4 as moderately severe (15–19 points), and 5 was identified as severe (20–27 points) [36].

#### 2.2.2. Eating Broadcast Viewing Experience

People who watched TV programs broadcasting cooking, consuming food, or talking about food on terrestrial (e.g., KBS, MBC, SBS), cable (e.g., Olive TV, tvN, ComedyTV, K STAR), or comprehensive programming (e.g., JTBC, TV CHOSUN, CHANNEL A, MBN) channels in the recent one-year had a TV eating broadcast viewing experience. People who watched uploaded videos broadcasting cooking, eating food, or talking about food on Korean popular online platforms such as YouTube and AfreecaTV in the recent one-year had an online eating broadcast viewing experience. In previous studies with reliable and validated scales such as Bergen Social Media Addiction Scale [37], Bergen Facebook Addiction Scale [38], and Mukbang Addiction Scale [39], experiences of media use were also measured by asking how often they used media during the last year. We used two questions to assess eating broadcast viewing experiences, and participants were divided into four groups: those who had both TV and online eating broadcast viewing experience, those who had TV eating broadcast viewing experience, those who had online eating broadcast viewing experience, and those who had no experience.

#### 2.2.3. Binge Drinking Frequency

The frequency of binge drinking soju and beer, which were the most popular alcoholic beverages in Korea, was measured to assess excessive alcohol use [40]. Binge drinking was described as drinking seven or more cups of soju (five or more cups of 355 cc beer cans) in one sitting for men and five or more cups of soju (three or more 355 cc beer cans) in one sitting for women [3]. The responses were coded on a frequency scale of 1 to 5:1 was identified none, 2 as less than once a month, 3 as about once a month, 4 as about once a week, and 5 identified as almost every day.

#### 2.2.4. Obesity-Related Eating Behaviors

The Dutch Eating Behavior Questionnaire (DEBQ) was used to assess obesity-related eating behaviors, which were classified as restrained eating, emotional eating, and external eating behaviors [41]. Restrained eating is a conscious choice and attempts to limit food consumption and calories to regulate body weight [42]. Emotional eating referred to a proclivity to eat in response to unpleasant feelings, such as sadness, disappointment, and isolation, while external eating referred to a proclivity to eat in response to external food stimuli, such as the sight, scent, and taste of food [42].

DEBQ was a survey tool with proven reliability and validity for assessing dietary type [41]. For restrained eating and external eating, the Korean DEBQ, which had previously been validated in 1996, was tested by health experts and used after modifications [43]. Emotional eating was assessed using the “clearly labeled motions scale” among the three original scale versions [41], which was translated into Korean by health experts. There were 10 items for restrained eating, 9 items for emotional eating, and 10 items for external eating. Each item was scored on a scale of 1 (never) to 5 (frequently), and the average score was computed (reverse coding the external eating questions). The Korean version of DEBQ used in this study had acceptable content validity through qualitative review by experts in health behavior change and health communication. Also, it showed high reliability with Cronbach’s alpha values of 0.906 for restrained eating behavior, 0.935 for emotional eating behavior, and 0.852 for external eating behavior.

### 2.3. Statistical Analysis

SPSS statistics software package version 23.0 was used for all analyses (SPSS Inc., Chicago, IL, USA). Demographic variables were analyzed using the analyses of variance (ANOVAs) and chi-squared tests by eating broadcast viewing experience. Welch’s ANOVAs were performed if the variable’s data violated the assumption of variance homogeneity [44]. When the ANOVA statistics were significant, Gabriel post hoc tests were performed, which are appropriate for unequal cell sizes. Dunnett T3 post hoc tests were performed when the statistics from Welch’s ANOVAs were significant, which is appropriate when the variances are unequal. We calculated Pearson correlations between binge drinking frequency and obesity-related eating behaviors. Binge drinking frequency and obesity-related eating behaviors were compared between groups of eating broadcast viewing experiences using ANOVA.

**Figure 1:**
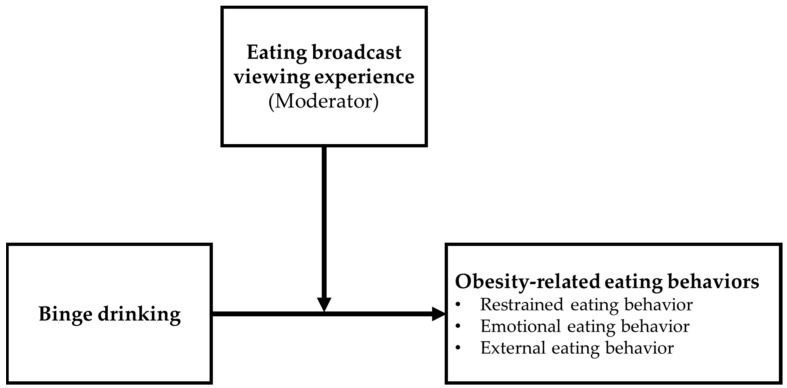
Conceptual model explaining obesity-related eating behaviors by binge drinking and eating broadcast viewing experiences.

Figure 1 depicts the conceptual model of eating broadcast viewing experience as a moderating role in the relationship between binge drinking frequency and obesity-related eating behaviors. Hayes’ PROCESS macro version 3.5 for SPSS was used to perform moderation analyses, which can compute moderation analyses using an ordinary least squares regression framework [45]. The moderating effects were estimated using the unstandardized regression coefficients (B), standard errors (SE), *t*-values, *p*-values, and lower (LLCI) and upper (ULCI) limit 95% confidence intervals for B. Since the eating broadcast viewing experience was a multi-categorical moderator, the results of the interaction between X and W and conditional effects of X on Y in the moderator’s categories were used to interpret statistical models.

Three moderation models were tested with restrained eating behavior, emotional eating behavior, and external eating behavior. Seven moderation models were additionally tested with the same variables in subgroups by sex (men and women) and age group (20s, 30s, 40s, 50s, and 60s–70s) for significant obesity-related eating behaviors. Before analyses, all continuous variables that defined products were mean-centered for all models. The PROCESS macro was also used to perform simple slope analyses and graphical displays to identify a significant interaction between binge drinking and each type of eating broadcast viewing experience on obesity-related eating behaviors.

## 3. Results

### 3.1. Demographics

Table 1 shows the sociodemographic characteristics and health status of the sample. Of the 1125 adults studied, 574 (51.02%) were men, and the age ranged from 20 to 73 years with a mean age of 42.46 years. More than half (56.00%) of the sample were married. The average household income was 5468.04 million won per year, and more than 90% of those polled lived in cities. Most participants were of normal weight after samples were categorized by BMI values, followed by obesity. The mean level of stress was 3.25 on a 5-point Likert scale, and the mean level of depression was 1.77 on the PHQ 9 scale, which indicates no depression to mildly severe. In terms of disease history, 683 (60.71%) individuals stated that they had not been diagnosed with hypertension, dyslipidemia, diabetes, cardiovascular disease, cancer, or depression.

People who had never experienced eating broadcasts and only watched TV eating broadcasts were older than those who watched both TV and online eating broadcasts (F = 11.845, *p* < 0.001). The distribution of age differed in terms of eating broadcast viewing experience, with the majority of those in their 20s watching both TV and online eating broadcasts and the majority of those in their 60s never having done so (χ^2^ = 58.839, *p* < 0.001). The average stress level was highest in people who watched eating broadcasts on TV and online (F = 4.121, *p* = 0.006). Only age and stress level were significant in the comparison by eating broadcast viewing experience, but all variables were used as covariates in the moderation analysis models.

### 3.2. Associations between Binge Drinking Frequency, Obesity-Related Eating Behaviors, and Eating Broadcast Viewing Experience

The mean values, standard deviations (SD), and Pearson correlation coefficients between binge drinking frequency and obesity-related eating behaviors are shown in Table 2. Binge drinking frequency was inversely related to restrained eating behavior and positively related to emotional eating and external eating behaviors.

Table 3 shows the mean differences in binge drinking frequency and obesity-related eating behaviors based on the eating broadcast viewing experience. Binge drinking frequency did not vary by eating broadcast viewing experience (F = 0.515, *p* = 0.672), but obesity-related eating behaviors did. People who viewed eating broadcasts online and on the TV had higher scores in restrained eating (F = 3.373, *p* = 0.018) behaviors than those who had never seen an eating broadcast. People who watched eating broadcasts on both the TV and online channels scored higher for emotional eating (F = 5.411, *p* = 0.001) and external eating (F = 7.769, *p* < 0.001) behaviors than those who never watched an eating broadcast and only watched TV broadcasts.

### 3.3. Moderation Effects of Eating Broadcast Viewing Experience on the Relationship between Binge Drinking Frequency and Obesity-Related Eating Behaviors

Interactions between binge drinking frequency (X) and eating broadcast viewing experience (W) had a significant effect on external eating behaviors (F_change_ = 2.686, *p* = 0.045, Model 1), but not on restrained eating (F_change_ = 2.092, *p* = 0.100) or emotional eating (F_change_ = 0.7949, *p* = 0.497) behaviors. Additional subgroup moderation analyses in terms of sex and age groups were conducted with external eating behavior. As a result of the analysis, the interaction between X and W was only significant for women (F_change_ = 7.133, *p* = 0.001, Model 2) and not men (F_change_ = 0.660, *p* = 0.577). The interaction between X and W affected only those in their 20s (F_change_ = 3.647, *p* = 0.014, Model 3), while those in their 30s (F_change_ = 1.087, *p* = 0.355), 40s (F_change_ = 1.846, *p* = 0.140), 50s (F_change_ = 1.260, *p* = 0.293), and 60s-70s (F_change_ = 1.221, *p* = 0.303) were unaffected.

**Figure 2:**
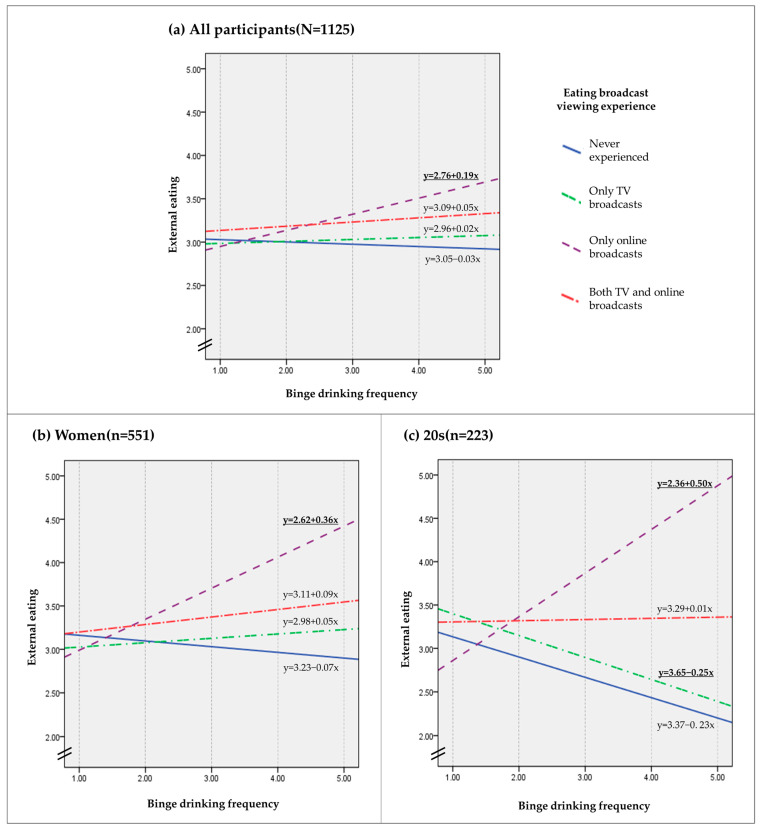
Conditional effects of binge drinking frequency on external eating behavior in four types of eating broadcast viewing experiences among (**a**) all participants, (**b**) women, and (**c**) individuals in their 20s controlled by sex, age group, marriage, annual household income, residence, BMI, stress level, depression level, and disease history. With a *p*-value of less than 0.05, the regression functions in bold and underlined were significantly different from zero.

Figure 2 displays the results of the moderation analysis for Models 1, 2, and 3. Each slope of the line produced by separate simple slope analyses represents the conditional effects of binge drinking frequency for different types of eating broadcast viewing experience. Covariates such as sex, age group, marriage, annual household income, residence, BMI, stress level, depression level, and disease history were used to control the results in Model 1. Model 2 had the same covariates as Model 1 except for sex, and Model 3 had the same covariates as Model 1 except for age group.

- Model 1 (Figure 2a): The full regression model accounted for 14.62% of the variance in external eating (F = 11.859, *p* < 0.001, R^2^ = 0.1462), with the inclusion of the interaction between X and W accounting for approximately 0.62% of the explained variance (R^2^ _change_ = 0.0062). According to the conditional effects, there was no association between binge drinking frequency and external eating among people who never watched eating broadcasts (B = −0.029, SE = 0.042, t = −0.688, *p* = 0.491, 95% LLCI = −0.112, 95% ULCI = 0.054), only watched eating broadcasts on TV (B = 0.024, SE = 0.029, t = 0.811, *p* = 0.417, 95% LLCI = −0.033, 95% ULCI = 0.080), or watched both TV and online eating broadcasts (B = 0.031, SE = 0.017, t = 1.877, *p* = 0.061, 95% LLCI = −0.001, 95% ULCI = 0.064). However, among participants who only viewed online eating broadcasts, an increase in binge drinking frequency was associated with an increase in external eating behaviors (B = 0.204, SE = 0.071, t = 2.875, *p* = 0.004, 95% LLCI = 0.065, 95% ULCI = 0.344).
- Model 2 (Figure 2b): The model was significant and accounted for 16.67% of the variance in external eating among women (F = 7.133, *p* < 0.001, R^2^ = 0.1667). Including the interaction between X and W improved the model fit (R^2^ _change_ = 0.0153). Separate simple slope analyses showed similar associations with Model 1. There was no association between binge drinking frequency and external eating among people who never experienced eating broadcasts (B = −0.121, SE = 0.083, t = −1.464, *p* = 0.144, 95% LLCI = −0.284, 95% ULCI = 0.041), who only watched eating broadcasts on TV (B = 0.048, SE = 0.054, t = 0.888, *p* = 0.375, 95% LLCI = −0.058, 95% ULCI = 0.153), or who watched such broadcasts on TV and online (B = 0.045, SE = 0.025, t = 1.805, *p* = 0.072, 95% LLCI = −0.004, 95% ULCI = 0.095). However, an increase in binge drinking frequency was associated with an increase in external eating behavior by women who only watched eating broadcasts online (B = 0.316, SE = 0.113, t = 2.809, *p* = 0.005, 95% LLCI = 0.095, 95% ULCI = 0.538).
- Model 3 (Figure 2c): The model was significant and accounted for 22.08% of the variance in external eating among those in their 20s (F = 3.911, *p* < 0.001, R^2^ = 0.2208). The model fit improved when the interaction between X and W was included (R^2^ _change_ = 0.041). Separate simple slope analyses indicated that, among those in their 20s who only viewed eating broadcasts online, an increase in binge drinking frequency was associated with an increase in external eating behaviors (B = 0.411, SE = 0.178, t = 2.314, *p* = 0.022, 95% LLCI = 0.061, 95% ULCI = 0.762). In contrast, among those in their 20s who only viewed eating broadcasts on TV, an increase in binge drinking frequency was associated with a decrease in external eating behaviors (B = −0.234, SE = 0.119, t = −1.975, *p* = 0.0496, 95% LLCI = −0.468, 95% ULCI = −0.0004). This association was not significant among people who never experienced eating broadcasts (B = −0.266, SE = 0.198, t = −1.341, *p* = 0.182, 95% LLCI = −0.656, 95% ULCI = 0.125) or who watched such broadcasts on the TV and online (B = 0.002, SE = 0.038, t = 0.043, *p* = 0.966, 95% LLCI = −0.073, 95% ULCI = 0.076).

## 4. Discussion

The purpose of this study was to examine the moderating effects of eating broadcast viewing experiences on the relationship between binge drinking frequency and obesity-related eating behaviors among Korean adults. The results indicate that the relationship between binge drinking and certain eating behaviors depended on the type of broadcast viewing. Among Korean adults, those who only watched online eating broadcasts had a significant moderating effect, particularly in women and those in their twenties (20s), because the more frequently they consumed excessive alcohol, the higher the level of external eating. Furthermore, people in their 20s who only watched eating broadcasts on TV had a negative association between binge drinking and external eating. This study implies that health problems caused by binge drinking should be considered with social and environmental elements, such as the media, rather than focusing solely on causality at the biological level.

There was no significant difference in binge drinking frequency depending on the experience of watching eating broadcasts in this study, but the interaction between the two affected external eating. This study used a moderation analysis to identify potential effects such as an interaction between binge drinking and watching eating broadcasts on unhealthy eating behaviors. In previous studies with college students and adolescents, there were no direct connections between alcohol consumption and social media use [46,47]. The experience of watching eating broadcasts could be related to cultural underlying factors related to binge drinking rather than to binge drinking itself. The influence of social norms and the sympathetic bond between content providers and consumers were the primary reasons watching eating broadcasts are popular [13,20]. Meanwhile, drinking alcohol is considered a social behavior in Korean society, and there is a culture of socializing by drinking with colleagues at work and peers at school [48,49,50]. The unique Korean drinking culture based on Korean sentiments needs to be considered when understanding the unusual popularity of watching eating broadcasts in Korea.

The interaction between binge drinking frequency and eating broadcast viewing experience was not related to attempts to limit food and eating due to unpleasant feelings, but rather to external food-related cues. Our results provided evidence that not only does the experience of watching eating broadcasts become external food stimuli, but so does the interaction with binge drinking. A recent study found that external eating and emotional eating both had positive and statistically significant indirect correlations with binge drinking, but restrained eating had a negative association [51]. Given that our findings regarding external eating were similar to this previous study, interventions targeting obesity-related eating behaviors and in binge drinkers should be shaped by external eating behaviors.

Conditional effects from the moderation analysis revealed that binge drinking frequency and the level of external eating were positively associated in people who had only seen online eating broadcasts. Eating broadcasts have become more common due to the recent success of single broadcasting media, and they are now largely produced and transmitted online [14]. In contrast to television, however, online media channels were free to regulate content. When this survey was conducted in 2019, television broadcasts were regulated for alcohol advertising under the National Health Promotion Act, but online broadcasts were not. As a result, online channels were more likely than TV to broadcast more aggressive content, including drinking alcohol, as a visual food stimulus that could induce external eating. In Korean eating broadcasts, unhealthy eating behavior scenes were identified more frequently in online broadcasts than on TV [15]. It was also reported that college students who used alcohol-related social media had a higher association of alcohol consumption and risky behaviors than those who used social media generally [46]. Our findings suggest that the influence of online eating broadcast content, particularly alcohol-related content, should be considered when assessing the obesity-related externality of binge drinkers.

Meanwhile, numerous neurobiological studies indicate that women and men show different brain activity patterns in response to visual food-related stimuli. When exposed to visual food cues while fasted, women have increased activity in the fusiform gyrus and frontal, striatal, and limbic areas of the brain compared to men, implying that women may be more sensitive to visual food stimuli, particularly when hungry [52]. Females also had higher levels of neural responses to food stimuli in the parahippocampus, precuneus, and thalamus than males according to a meta-analysis [53]. Given that many recent neuroimaging findings showed that binge drinking can affect brain development, these were notable results [54]. To summarize, amid the growing problem of binge drinking among Korean women, the rising popularity of online eating broadcasts may impact women’s external eating-related characteristics.

People in their 20s who watch online eating broadcasts had more external eating habits associated with more frequent binge drinking. This moderation effect for people in their 20s was the largest among the significant moderation models. According to the results of the 2019 National Health and Nutrition Survey in Korea, the monthly binge drinking rate among people in their 20s (48.0%, ±2.1) was the highest among all age groups. The monthly binge drinking rate of women in their 20s was 44.1% (±3.1), which was 17.9% higher than those in their 30s, who were the second-highest age group [2]. Among the subjects in this study, the binge drinking frequency for those in their 20s was the second-highest rate, after those in their 30s, and women in their 20s were remarkably more frequent binge drinkers than other age groups. Despite some differences, the results from national data and study data were similar. Since exposure to social media influences social norms and behaviors in young adults [55], watching online eating broadcasts is likely to influence some eating behaviors in Koreans in their 20s with serious binge drinking issues. In conclusion, the health of those in their 20s, who are transitioning into adulthood, is important due to the socio-economic aspects of health care burdens. This study of the unhealthy eating behavior of those in their 20s involving binge drinking and new forms of media is noteworthy.

What distinguishes those in their 20s who have only watched TV eating broadcasts is that the more frequently they binge drink, the lower their level of external eating. This raises the possibility that watching TV eating broadcasts may protect viewers against the unhealthy relationships between binge drinking frequency and external eating. A study found that watching TV did not always have a direct negative effect on eating disorder symptoms [26], and another found that watching social media and TV resulted in a better debate than watching TV alone [56]. However, because this study was cross-sectional, it is unreasonable to expect binge drinkers in their 20s, particularly those who watch online eating broadcasts, to reduce their external eating by encouraging them to watch only TV eating broadcasts. Consequently, as previous research revealed that problem drinking was associated with other health risk behaviors globally [57,58], it is necessary to examine whether people in their 20s who watched only TV eating broadcasts frequently binge-drank, resulting in relatively less external eating due to other problematic eating or other unhealthy behaviors.

There were several study limitations. First, because this study analyzed cross-sectional data, we could not prove a causal relationship based on the conceptual model. Second, since the researcher arbitrarily designated binge drinking as a continuous variable, the frequency of binge drinking was measured on a five-point scale, which may have caused bias. Third, the validity of the Korean emotional eating DEBQ scale is questionable because we did not use a reverse translation process. We did try to reduce errors by translating the existing Korean DEBQ and having health and behavior experts review the scale. Fourth, the participants who only watched online eating broadcasts may not be representative due to the small sample size (39 people). Further larger sample size studies are needed to replicate and confirm our findings. However, given that the majority of people watched both online and TV eating broadcasts, the number differences between the groups can also help interpret the results. Fifth, the experience of watching eating broadcasts can be classified not only by media type, but also by viewing time, time zone, and people watching them together. Lastly, while this study did not examine whether drinking behavior is part of external eating, future research will differentiate between external eating and drinking behavior based on external stimuli. As several studies have linked social media alcohol content with problem drinking [59,60], it is important to investigate the potential risks of drinking exposure on eating broadcasts. In terms of national policy in Korea, the media covered by the alcohol advertising ban (7 a.m. to 2 p.m.) was expanded to include data and internet multimedia broadcasting [61]. This will go into effect on 30 June 2021, making it possible to compare the longitudinal impact.

Nevertheless, the present finding suggests that the impact of serious problem drinking behaviors in certain societies needs to be considered in conjunction with their interactions with the media environment. Specifically, the interaction between binge drinking and eating broadcast viewing experience should be assessed for healthy eating. Results of this research can be applied to identify and prioritize target groups for interventions addressing eating behaviors and obesity in binge drinkers, as well as to develop intervention content. Women or those in their 20s who only watched online eating broadcasts can be considered as the priority target group for the intervention. Given the impulsive and addictive characteristics of binge drinkers, interventions that include educational content must be developed to help them recognize the influence of eating broadcasts as a food-related cue that would provoke external eating in the real world. According to a study on Korean online eating shows, the staff and producers need to have a social responsibility to warn viewers of the harmful effects of unhealthy foods, provide education regarding a balanced diet for obesity prevention, and encourage healthy viewing habits to mitigate the negative impact of these programs [56]. In this context, health education programs based on national alcohol policy should take into account the negative impact of the media environment, particularly eating broadcasts, on the health problems and behaviors of Korean drinkers who live in a culture that promotes binge drinking.

## 5. Conclusions

Binge drinking has recently become a serious problem in Korea, especially for women and those in their 20s. Meanwhile, eating broadcasts from Korea are gaining worldwide popularity. Binge drinking can be influenced by the environment and are related to eating behaviors that induce obesity. The goal of this study was to examine whether eating broadcast viewing experiences affected the association between the frequency of binge drinking and obesity-related eating behaviors among Korean adults. This is the first study, to our knowledge, to investigate the influence of eating broadcast viewership as an environmental factor related to binge drinking.

The relationship between binge drinking frequency and external eating among Koreans was dependent on how they watched eating broadcasts. The interaction between binge drinking and eating broadcasts viewing is one external food-related stimuli. According to the conditional effects of moderation models analyzed in this paper, subjects who viewed eating broadcasts by online platforms only had more external eating with more frequent binge drinking. This tendency appeared among women and those in their 20s. This is most likely connected to their binge drinking and how they respond to social media or food-related cues. Interestingly, more frequent binge drinking results in lower levels of external eating in people in their 20s who watched only TV eating broadcasts. This implies that watching TV eating broadcasts may have a protective effect against external eating among binge drinkers.

To understand the detrimental effects of binge drinking on obesity-related eating behaviors, this study suggests watching broadcasts as an environmental factor associated with binge drinking. Future research should consider various aspects of the eating broadcast viewing experience, media type, and how TV and online eating broadcasts content, particularly those that involve drinking alcohol, affect binge and problem drinking. To prevent the negative effects of eating broadcasts on eating behaviors and obesity-related health problems, the government should provide educational support.

## Figures and Tables

**Table 1:** Sociodemographic characteristics and health status for study variables by eating broadcast viewing experience.

Variables	Mean (±SD) or *n* (%)					F or χ^2^	Post-Hoc Analysis
	Total (*n* = 1125)	1	2	3	4		
		Never Experienced (*n* = 96)	Only TV Broadcasts (*n* = 220)	Only Online Broadcasts (*n* = 39)	Both TV and Online Broadcasts (*n* = 770)		
Sex							
Men	574 (51.02%)	59 (61.46%)	116 (52.73%)	20 (51.28%)	379 (49.22%)	5.441	
Women	551 (48.98%)	37 (38.54%)	104 (47.27%)	19 (48.72%)	391 (50.78%)		
Age (years)	42.46 (±13.52)	45.72 (±14.34)	46.31 (±12.43)	42.36 (±15.12)	40.95 (±13.36)	11.845 ***^2^	1 > 4, 2 > 4 ^3^
20s	223 (19.82%)	13 (13.54%)	19 (8.64%)	7 (17.95%)	184 (23.90%)	58.839 ***	
30s	336 (29.86%)	29 (30.21%)	58 (26.36%)	15 (38.46%)	234 (30.39%)		
40s	229 (20.36%)	17 (17.71%)	52 (23.64%)	4 (10.26%)	156 (20.26%)		
50s	109 (9.69%)	7 (7.29%)	39 (17.73%)	2 (5.13%)	61 (7.92%)		
60s	226 (20.09%)	30 (31.25%)	52 (23.64%)	11 (28.21%)	133 (17.27%)		
70s	2 (0.18%)	0 (0.00%)	0 (0.00%)	0 (0.00%)	2 (0.26%)		
Marriage							
Single	495 (44.00%)	37 (38.54%)	89 (40.45%)	20 (51.28%)	349 (45.32%)	3.671	
Married	630 (56.00%)	59 (61.46%)	131 (59.55%)	19 (48.72%)	421 (54.68%)		
Annual household income [per 10.000 won]	5468.04 (**±**3912.20)	4956.77 (±3584.62)	5042.94 (±2783.91)	6849.23 (±9101.36)	5583.29 (±3780.04)	2.572 ^2^	
Residence							
Urban areas	1020 (90.67%)	86 (89.58%)	190 (86.36%)	35 (89.74%)	709 (92.08%)	6.798	
Rural areas	105 (9.33%)	10 (10.42%)	30 (13.64%)	4 (10.26%)	61 (7.92%)		
BMI (kg/m^2^)	23.29 (**±**3.59)	23.62 (±3.71)	23.47 (±3.54)	24.18 (±3.39)	23.15 (±3.60)	1.623	
Underweight	70 (6.22%)	7 (7.29%)	13 (5.91%)	2 (5.13%)	48 (6.23%)		
Normal weight	495 (44.00%)	34 (35.42%)	88 (40.00%)	14 (35.90%)	359 (46.62%)		
Overweight	240 (21.33%)	26 (27.08%)	56 (25.45%)	7 (17.95%)	151 (19.61%)		
Obesity	320 (28.44%)	29 (30.21%)	63 (28.64%)	16 (41.03%)	212 (27.53%)		
Stress level ^1^	3.25 (**±**0.73)	3.14 (±0.83)	3.12 (±0.75)	3.23 (±0.78)	3.30 (±0.71)	4.121 **	2 < 4 ^4^
Depression level ^1^	1.77 (**±**0.94)	1.91 (±1.15)	1.66 (±0.92)	1.87 (±1.08)	1.78 (±0.91)		
Disease history							
None	683 (60.71%)	56 (58.33%)	130 (59.09%)	20 (51.28%)	477 (61.95%)	2.417	
Diagnosed	442 (39.29%)	40 (41.67%)	90 (40.91%)	19 (48.72%)	293 (38.05%)		

** *p* < 0.01, *** *p* < 0.001, ^1^ Range from 1–5, ^2^ Welch’s ANOVA results, ^3^ Dunnett T3 post-hoc test results, ^4^ Gabriel post-hoc test results. SD: Standard deviation.

**Table 2:** Correlations among binge drinking frequency and obesity-related eating behaviors.

Variables		Pearson Correlation Coefficients			
		1	2	3	4
1	Binge drinking frequency	1			
2	Restrained eating behavior (average score)	−0.086 **	1		
3	Emotional eating behavior (average score)	0.061 *	0.165 **	1	
4	External eating behavior (average score)	0.091 **	−0.041	0.462 **	1

* *p* < 0.05, ** *p* < 0.01.

**Table 3:** Binge drinking frequency and obesity-related eating behaviors by eating broadcast viewing experience.

Variables	Mean (±SD) or n (%)					F	Post-Hoc Analysis
	Total (n = 1125)	1	2	3	4		
		Never Experienced (n = 96)	Only TV Broadcasts (n = 220)	Only Online Broadcasts (n = 39)	Both TV and Online Broadcasts (n = 770)		
Binge drinking frequency	2.15 (1.24)	2.02 (1.31)	2.12 (1.29)	2.21 (1.24)	2.18 (1.22)	0.515	
Restrained eating behavior (average score)	3.04 (0.71)	2.90 (0.75)	2.96 (0.67)	3.00 (0.77)	3.09 (0.71)	3.373 *	1 < 4 ^1^
Emotional eating behavior (average score)	2.40 (0.85)	2.21 (0.78)	2.25 (0.84)	2.38 (0.91)	2.46 (0.84)	5.411 **	1 < 4, 2 < 4 ^1^
External eating behavior (average score)	3.14 (0.58)	3.00 (0.54)	3.01 (0.55)	3.17 (0.67)	3.19 (0.58)	7.769 ***	1,2 < 4 ^1^

* *p* < 0.05, ** *p* < 0.01, *** *p* < 0.001, ^1^ Gabriel post-hoc test results. SD: Standard deviation.

## Data Availability

Not applicable.
